# Insufficient Sleep and Poor Sleep Quality Completely Mediate the Relationship between Financial Stress and Dietary Risk among Higher Education Students

**DOI:** 10.3390/bs11050069

**Published:** 2021-05-05

**Authors:** Chen Du, Wenyan Wang, Pao Ying Hsiao, Mary-Jon Ludy, Robin M. Tucker

**Affiliations:** 1 Department of Food Science and Human Nutrition, Michigan State University, East Lansing, MI 48824, USA; duchen@msu.edu (C.D.); wangwe60@msu.edu (W.W.); 2 Department of Food and Nutrition, Indiana University of Pennsylvania, Indiana, PA 15705, USA; pyhsiao@iup.edu; 3 Department of Public and Allied Health, Bowling Green State University, Bowling Green, OH 43403, USA; mludy@bgsu.edu

**Keywords:** sleep, sleep quality, sleep duration, diet, dietary intake, mediation analysis, COVID-19

## Abstract

The coronavirus disease 2019 (COVID-19) pandemic worsened financial stress for higher education students in the U.S. Financial stress is associated with poor dietary behaviors; however, factors that might influence this relationship are not well characterized. The present cross-sectional study investigated the associations between financial stress and dietary intake and dietary risk scores among higher education students (undergraduate and graduate students) in the U.S. and examined whether poor sleep quality and short sleep duration mediated the relationship between financial stress and dietary risk score. Validated tools were used to assess financial stress, sleep quality, sleep duration, dietary intake, and dietary risk. A total of 1280 students from three large U.S. universities completed the study. Results indicated that higher financial stress was associated with lower vegetable, fruit, fiber, and calcium intake, higher added sugar intake from sugar sweetened beverages, and higher dietary risk score. Further, the positive relationship between financial stress and dietary risk score was completely mediated by poor sleep quality among students who reported poor sleep quality and by short sleep duration among students who slept less than 7 h per night. These findings suggest that students might benefit from both financial management training and sleep education services to reduce undesirable dietary behaviors.

## 1. Introduction

Financial stress, characterized as difficulty meeting financial obligations [1], is highly prevalent among higher education students (both undergraduate and graduate) in the U.S., and the recent pandemic has exacerbated financial stress in this population [2]. Financial concerns have been frequently cited by U.S. students as stressful or as reasons for contemplating leaving school [3,4,5]. The Spring 2019 National College Health Assessment reported more than one-third of college students stated that their finances had been traumatic or very difficult to handle during the past 12 months [6]. Unfortunately, the COVID-19 pandemic further worsened the financial situation for many students in higher education [7,8]. The National College Student COVID-19 Survey revealed that 30% of students reported losing a job needed to help pay for college, 77% of students reported that the pandemic reduced their ability to earn income to support their education, and 64% stated that their need for financial aid increased due to the pandemic [9]. Based on the available evidence, an overwhelming number of higher education students in the U.S. face financial stress.

Financial stress is associated with various negative health behaviors, including poor dietary behaviors, which can lead to undesirable health outcomes [10,11,12,13]. Studies among higher education students report that greater financial difficulties are associated with skipping breakfast [10], frequent fast-food consumption [10], and restrained eating [13]. Additionally, previous work notes that the cost of food is one of the primary factors for young people in determining food selection [14]. For example, the cost of produce is the most frequently reported barrier preventing students from consuming fruit and vegetables [15]. Thus, greater financial stress is associated with more undesirable dietary behaviors. Further, poor dietary behaviors lead to undesirable health outcomes such as being overweight or obese [16,17], having a higher risk of suffering from chronic diseases [18,19,20], and having a greater likelihood of developing mental health problems [21,22]. In terms of mental health, recent research suggests adults who have been financially disadvantaged by the pandemic are experiencing higher levels of psychological distress than those who have not [23]. Based on the available evidence, the pandemic has placed increased financial stress on many students, and this increased stress is likely to also negatively affect dietary behaviors and health outcomes.

The current literature shows that financial stress associated with poor dietary behaviors, but these dietary behaviors are not well characterized among higher education students. Additionally, current investigations into the associations between financial stress and dietary behaviors focus on cost, but the cost of food is not easily modifiable. To our knowledge, investigation into other factors besides cost that could influence the relationship between financial stress and dietary behaviors has not been conducted. Therefore, the present study investigated other factors that could influence the relationships between financial stress and dietary intake and behaviors. These factors included sleep duration and quality.

Stress, including financial stress, can lead to short sleep duration and poor sleep quality [24,25,26,27], and these sleep problems can lead to poor dietary behaviors [28,29,30,31]. Thus, sleep may serve as a factor connecting financial stress and poor dietary behaviors. Several studies among university students report that higher financial stress scores or a greater number of financial strains predict lower sleep quality [24,32]. Further, reducing stress can improve both sleep duration and quality [26,27]. In terms of sleep and dietary intake, numerous studies note that short sleep duration and poor sleep quality increase undesirable dietary behaviors, such as: more frequent consumption of sweets [33], poor dietary quality [30,31], and increased total energy intake [29]. Given the relationships between financial stress and sleep and the relationships between sleep and dietary behaviors, sleep may serve as a mediator in these relationships.

To address the current knowledge gaps and to better understand the complex relationships between financial stress, sleep, and dietary behaviors, the present study: (1) examined the associations between financial stress and dietary intake and risky dietary behaviors, and (2) investigated whether short sleep duration and poor sleep quality mediated the relationship between financial stress and dietary risk. Based on the documented relationships between financial stress, sleep, and dietary behaviors, hypotheses included:

1) A higher financial stress score would be associated with a lower intake of healthy food groups and nutrients and a higher intake of unhealthy food groups and nutrients.
2) A higher financial stress score would be associated with a higher overall dietary risk score.
3) Poor sleep quality, but not good sleep quality, would mediate the relationship between financial stress and overall dietary risk score.
4) Short sleep duration, but not adequate sleep duration, would mediate the relationship between financial stress and overall dietary risk score.

## 2. Materials and Methods

### 2.1. Study Population and Design

The population of the present study was a sub-sample of a multi-country and multi-institute cross-sectional study [34]. The details of the primary study, including study population and design were published previously [34]. In short, undergraduate and graduate students who were at least 18 years old and were studying at universities in China, Ireland, Malaysia, Taiwan, South Korea, the Netherlands, and the U.S. were recruited in the primary study. Only students studying at U.S. universities were included in the present study because the dietary questionnaires used in the present study were only validated in the U.S. population. The students in the present study were recruited from three large universities in Michigan, Ohio, and Pennsylvania. Eligible students filled out online surveys delivered through Qualtrics (SAP, Provo, Utah, USA) from April to the end of May 2020 during which time all three states involved in the study were under “shelter in place” orders. Informed consent was obtained from all subjects involved in the study.

### 2.2. Measures

#### 2.2.1. Demographics and Anthropometrics

Eligible students self-reported age, gender, class status (undergraduate vs. graduate), residency status (domestic vs. international), weight and height in the surveys. Body mass index (BMI) was calculated using the self-reported weight and height information.

#### 2.2.2. Financial Stress

Financial stress was assessed using the validated University Student Financial Stress Assessment (USFSA) tool, which is a 6-item questionnaire included in the 2010 Ohio Student Financial Wellness Survey (OSFWS) [35]. Questions 1 through 3 assess stress related to one’s personal financial situation, such as being able to pay monthly expenses or being able to pay for school. Responses for these three questions range from 1 (strongly disagree) to 4 (strongly agree). Questions 4 through 6 assess stress related to the total amount of money owed, credit card debt, and student loan debt. Responses for these three questions range from 1 (does not apply/no debt) to 6 (extreme amount). The total score of the USFSA ranges from 6 to 30 with higher scores indicating higher financial stress. Participants were also asked whether their financial stress was greater, less, or unchanged during the COVID-19 pandemic compared to before the pandemic.

#### 2.2.3. Perceived Stress

Perceived stress was assessed using the validated Perceived Stress Scale-10 (PSS-10) [36]. The PSS-10 questionnaire included 10 items, and responses for each item range from 0 (never) to 4 (very often). Example questions are “In the last month, how often have you been upset because of something that happened unexpectedly?”, and “In the last month, how often have you found that you could not cope with all the things that you had to do?” The total score of the PSS-10 ranges from 0 to 40, with higher scores indicating more perceived stress.

#### 2.2.4. Sleep Quality and Duration

Subjective sleep quality was assessed using the validated Pittsburgh Sleep Quality Index (PSQI) [37,38,39,40,41]. The PSQI consists of 10 main questions. The first four questions assess sleeping habits and duration; the other questions assess the degree of sleep problems. The PSQI scores range from 0 to 21, with higher scores indicating poorer sleep quality. In addition, participants were classified as poor sleepers if they reported a PSQI score of ≥5 [41]. Sleep duration on weekdays and weekends were collected by asking participants how many hours they usually sleep during the weekdays and weekends. Average sleep duration was calculated using a weighted method (((weekday sleep duration × 5) + (weekend sleep duration × 2))/7). Participants were classified as having short sleep duration if they reported a sleep duration <7 h per sleep duration guidelines [42].

#### 2.2.5. Dietary Intake

Dietary intake of specific food groups and nutrients was assessed using the Dietary Screener Questionnaire (DSQ) from the National Health and Nutrition Examination Survey (NHANES) 2009 to 2010 [43]. Dietary intake data were converted from intake frequencies and processed per the DSQ Data Processing and Scoring protocol [44]. The DSQ provides estimations of predicted intake of dairy (cup equivalents per day), whole grains (ounce equivalents per day), vegetables including legumes and excluding French fries (cup equivalents per day), fruit (cup equivalents per day), fiber (grams per day), calcium (milligrams per day), predicted intake of total added sugars (teaspoons equivalents per day), and predicted intake of added sugars from sugar-sweetened beverages (SSB, teaspoons equivalents per day) for males and females. The prediction equations used per protocol only calculate intake for males and females; thus, students who identified as “other” were not included in the analysis. The DSQ also measures frequency of processed meat and red meat consumption. Computation of consumption frequency does not require prediction equations; therefore, frequency of processed meat and red meat consumption was computed for all gender categories.

#### 2.2.6. Dietary Risk

Dietary risk score was assessed using a validated eight-item simplified food frequency questionnaire, Starting the Conversation (STC) [45]. The STC survey measures eating frequencies of fast foods, fruit, vegetables, soda and sweet tea, high quality proteins, chips and crackers, dessert, and solid fat. A sample question from the STC is “How many times a week did you eat fast food meals or snacks?” Answers for eating frequencies of low nutritional quality foods range from 0 (less than 1 time) to 2 (4 or more times) while answers for eating frequencies of high nutritional quality foods range from 0 (5 or more) to 2 (2 or less). Summing all answers provides a global score of dietary risk behaviors ranging from 0 to 16, with higher scores indicating more frequent engagement in dietary behaviors that are considered unhealthy.

#### 2.2.7. Physical Activity

Physical activity was measured using the validated International Physical Activity Questionnaire (IPAQ) long form [46]. Participants reported walking, moderate, and vigorous activities in terms of frequency and duration for five domains of activity including occupation-related, transportation, housework, walking, and recreation. Activities for each domain were calculated per the IPAQ long form protocol [47] and were reported using metabolic equivalents (METs) minutes per week. The total METs minutes per week were the sum of the activities for each domain. Physical activity was assessed in order to determine if it should be considered a covariate in the analyses.

#### 2.2.8. Mediation Models

**Figure 1:**
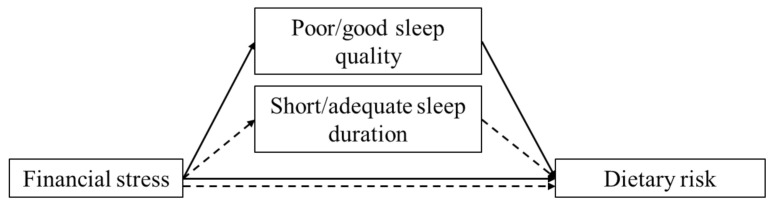
Proposed mediation models. The effects of financial stress on dietary risk scores through the mediation of sleep quality among poor sleepers (mediation model 1, solid line) or good sleepers (mediation model 3, solid line). The effects of financial stress on dietary risk scores through the mediation of sleep duration among students who reported short sleep duration (mediation model 2, dashed line) or students who reported adequate sleep duration (mediation model 4, dashed line).

To test whether poor sleep quality and short sleep duration mediated the relationship between financial stress and dietary risk scores, two models were built (Figure 1). Participants who had a PSQI score ≥5 were included in mediation model 1, and participants who reported sleeping <7 h were included in mediation model 2. To test whether sleep quality and duration also mediate the relationship between financial stress and dietary risk scores among “good” sleepers, the same analyses were run among students who reported good sleep quality (PSQI < 5) (model 3) and who reported meeting the minimum recommended sleep duration of 7 h per day (model 4).

The dietary risk score (STC score) presents a global score for unhealthy dietary behaviors; therefore, they were used in the mediation models. Dietary intake was not examined in the models because there was no global score that could be used to represent total intake.

### 2.3. Statistical Analyses

Descriptive statistics were performed and were presented as means ± standard deviations (SD) for continuous variables and percentages (%) for categorical variables. Bivariate correlation analyses were conducted using Pearson correlation tests or one-way ANOVA. The purposes of the bivariate correlation analyses were to assess the relationships between the independent variable (financial stress) and covariates (age, gender, graduate status, residency status, BMI, sleep quality, sleep duration, physical activity, and perceived stress) and between the dependent variables (dietary intakes and the overall dietary risk score) and covariates. False discovery rate adjustment was performed; specifically, Benjamini–Hochberg adjusted *p* values were used to evaluate significance for the bivariate analyses for all continuous variables [48].

Multivariate regression analyses were conducted to assess the relationships between financial stress score and dietary intake and between financial stress score and dietary risk score. Statistical assumptions of multivariate regression analyses were tested, including normality, linearity, multicollinearity, and homoscedasticity. The sample data met all assumptions. Covariates adjusted in the models were evaluated conceptually and empirically [49,50]. Model A of the multivariate regression analysis represents the unadjusted model. Age, gender, and residency status were adjusted in model B, and model C further adjusted for perceived stress in addition to the variables adjusted in model B.

Mediation analyses were conducted to examine whether sleep quality and sleep duration mediated the relationship between financial stress and dietary risk scores. The mediation analyses were carried out in students who reported poor sleep quality, good sleep quality, short sleep duration, and adequate sleep duration separately. This approach allows for the determination of whether these variables individually mediate the relationship between financial stress and dietary risk scores and whether the mediating relationships are only present among students who reported poor sleep quality or short sleep duration. Power analysis was based on 20 participants per construct in each model; therefore, a minimum of 60 participants were needed in each model [51]. The analyses were conducted using the SPSS PROCESS Macro, and Model 4 was chosen for the analyses [52]. Age, gender, residency status, and perceived stress were adjusted in all four models. The number of bootstraps performed for bias corrected bootstrap confidence intervals was set at 10,000. To interpret the results of the mediation analyses, the indirect effect of an independent variable on a dependent variable through the mediator (bootstrap results) must be significant in order to show a significant mediation effect. Additionally, the singular direct effects of an independent variable on a dependent variable, an independent variable on a mediating variable, or a mediating variable on a dependent variable do not need to be significant in order for the mediation effect to be significant [52,53].

The DSQ data were prepared using SAS 9.4 (SAS Institute, Cary, NC, USA) and all the analyses were performed in SPSS version 26 (IBM Corporation, Armonk, NY, USA). The threshold for statistical significance was set at *p* < 0.05.

## 3. Results

### 3.1. Demographic and Anthropometric Information

A total of 1433 students initiated the survey; 1280 students completed the survey and were included in the analyses (Table 1). The majority of the students were female, undergraduate, and domestic students. The average BMI of the students was classified as overweight. The majority of the students reported poor sleep quality while most of the students reported meeting the minimum sleep recommendation of 7 h per night.

### 3.2. Associations between Financial Stress and Demographic and Health Characteristics

The majority of the students (52.8%) reported having greater financial stress during the COVID-19 pandemic compared to before, 8.8% of students reported less financial stress, and 38.4% of students reported no change in financial stress. Greater financial stress was associated with older age, and female students reported greater financial stress compared to male students (Table 2). Additionally, domestic students reported greater financial stress compared to international students. In terms of health characteristics, greater financial stress was associated with higher BMI, higher perceived stress, lower sleep quality, and shorter sleep duration.

### 3.3. Associations between Demographic and Health Characteristics and Dietary Intakes and Behaviors

Table 3 lists the Pearson product moment correlation values for demographic, perceived stress, sleep, and physical activity measures. Age and BMI were associated with dietary intakes and dietary risk score. Older age was associated with higher intake of whole grains, vegetables, fiber, total added sugar, and added sugar from SSB. In addition, older age was also associated with a lower dietary risk score. Higher BMI was associated with lower intake of whole grains, fruit, and fiber but with higher intake of red meat, processed meat, total added sugar, and added sugar from SSB. Higher BMI was also associated with a higher dietary risk score.

Higher perceived stress was associated with lower intake of dairy, whole grains, vegetables, fruit, red meat, fiber, and calcium but higher intake of total added sugar and added sugar from SSB. Further, higher perceived stress was associated with a higher dietary risk score.

Differences between the associations of sleep quality and duration with dietary intakes and dietary risk score were observed. Poorer sleep quality was associated with lower intake of whole grains, vegetables, fruit, fiber, calcium, and higher intake of total added sugars and added sugar from SSB. In addition, poorer sleep quality was associated with a higher dietary risk score. Shorter sleep duration was only associated with higher intake of added sugar from SSB.

Higher physical activity level was associated with higher intake of vegetables, fruit, fiber, and calcium. Furthermore, a higher level of physical activity was associated with a lower dietary risk score.

Female students reported lower intake of most foods except whole grains compared to male students, but the dietary risk scores measured using the STC did not differ between female and male students (Table 4). Compared to graduate students, undergraduate students consumed lower amounts of vegetables, fruits, and fiber, and reported higher dietary risk scores. Compared to international students, domestic students reported lower intake of vegetables, red meat, and fiber, and higher consumption of processed meat. Additionally, higher dietary risk scores were observed among domestic compared to international students.

### 3.4. Unadjusted and Adjusted Findings

The results of the unadjusted models indicated that higher financial stress was associated with lower vegetable, fruit, fiber, and calcium intake; higher added sugar intake from SSB; and higher dietary risk scores (Table 5). When adjusted for age, gender, and residency status, the results showed that higher financial stress was no longer associated with calcium intake, but the other significant relationships in the unadjusted model remained. After the adjustment for perceived stress in addition to age, gender and residency status, the association between financial stress and dietary risk score was no longer significant, but all other relationships remained.

### 3.5. Mediation Analysis

The mediation analysis of model 1, which examined poor sleepers, showed that financial stress and dietary risk score were not directly correlated, but they were indirectly correlated through poor sleep quality. This finding indicates that poor sleep quality completely mediated the relationship between financial stress and dietary risk score among students with poor sleep quality (Table 6). Additionally, higher financial stress was associated with poorer sleep quality, and poorer sleep quality was associated with a higher dietary risk score. However, the mediation effect of sleep quality on the relationship between financial stress and dietary risk score among students who reported good sleep quality was not significant (Table 7). Further, financial stress was not associated with sleep quality among students who reported good sleep quality.

The mediation analysis of model 2, which analyzed only short sleepers (<7 h per night), showed that financial stress and dietary risk score were not directly correlated, but they were indirectly correlated through short sleep duration. This result indicates that short sleep duration completely mediated the relationship between financial stress and overall dietary risk score among students who did not meet sleep recommendations (Table 8). Additionally, higher financial stress was associated with shorter sleep duration, and shorter sleep duration was associated with higher dietary risk scores. However, the mediation effect of sleep quality on the relationship between financial stress and dietary risk score among students who reported meeting minimum sleep recommendations was not significant (Table 9). Finally, financial stress was not associated with sleep duration among students who reported adequate sleep duration.

Overall, the mediation analyses demonstrated that the relationship between financial stress and dietary risk score was completely mediated by sleep quality among students who reported poor sleep quality and completely mediated by sleep duration among students who reported sleeping <7 h per night. This result indicates that the relationship between financial stress and overall dietary risk score was associated with poor sleep quality among poor quality sleepers and short sleep duration among short sleepers. Sleep quality and duration did not mediate the relationships between financial stress and dietary risk score for students reporting adequate or good quality sleep.

## 4. Discussion

The purpose of this study was to explore relationships between financial stress, dietary intake and behavior, and if sleep quality and duration mediated these relationships. As hypothesized, higher financial stress among higher education students was associated with lower vegetable, fruit, fiber, and calcium intake, higher added sugar intake from SSB, and higher dietary risk score. Further, the positive relationship between financial stress and dietary risk score was associated with both poor sleep quality among students who reported poor sleep quality and short sleep duration among students who did not meet the recommendation of at least 7 h of sleep per night. These results suggest that reducing financial stress and improving sleep quality and duration among higher education students could be effective strategies for improving dietary behaviors. These conclusions need further testing due to the cross-sectional nature of the present study.

Even though the relationships between financial stress and calcium intake and dietary risk scores disappeared after certain covariate adjustments, the lack of relationship after adjustment indicates that the covariates in question were significantly associated with these variables. For example, when adjusted for age, gender, and residency status, the results showed that higher financial stress was no longer associated with lower calcium intake. This finding indicates that these demographic covariates were associated with financial stress and calcium intake, but it should not be interpreted that there is no association between financial stress and calcium intake [54,55]. Additionally, after the adjustment of perceived stress in addition to age, gender, and residency status, the association between financial stress and dietary risk score was no longer significant. This indicates that perceived stress was associated with financial stress and dietary risk scores, but it should not be interpreted that no association between financial stress and dietary risk scores exists [54,55]. In summary, age, gender, and residency status were significant covariates in the relationship between financial stress and calcium intake while perceived stress was a significant covariate in the relationship between financial stress and dietary risk scores. Based on the bivariate correlations, gender was associated with calcium intake, and perceived stress was associated with both financial stress and dietary risk score. While the adjustment for covariates led to a loss of statistical significance, these findings do not indicate that there are no associations between financial stress and calcium intake or financial stress and dietary risk score.

### 4.1. Associations between Financial Stress and Dietary Intake and Dietary Risk Score

Stress, including financial stress, affects college students’ eating habits, attitudes, and behaviors [10,12,13,15,56,57]. The present study found that higher financial stress among higher education students was associated with lower vegetable, fruit, fiber, and calcium intake; higher added sugar intake from SSB; and higher overall dietary risk score. The findings are consistent with previous studies showing higher financial stress is associated with more frequent unhealthy eating behaviors among higher education students [10,56,58,59,60]. Students tend to reduce their food purchasing budget when they experience economic hardship [12], and healthy foods, such as fruit and vegetables, are perceived as high-cost food items [15]. Further, lower fiber intake and calcium intake are associated with lower fruit and vegetable intake [61,62], which could further explain our findings regarding the association between higher financial stress and lower fiber and calcium intake. Given the evidence previously published and the current results, higher financial stress is associated with lower diet quality and more frequent engagement in risky dietary behaviors.

Contrary to our hypothesis, financial stress was not associated with lower dairy, higher red meat, processed meat, and total sugar intake. These findings suggest that factors other than financial concerns play a more important role in consumption of these items. Further study is needed to determine which other factors contribute to these consumption decisions.

### 4.2. The Mediation Effect of Poor Sleep Quality on the Relationship between Financial Stress and Overall Dietary Risk Score

As hypothesized, the present study found that poor sleep quality mediated the relationship between financial stress and dietary risk score among students who reported poor sleep quality. This finding indicates that greater financial stress may disrupt sleep by lowering sleep quality, and poor sleep quality is associated with more frequent engagement in undesirable dietary behaviors. Financial stress is a leading stressor among higher education students that contributes significantly to their overall perceived stress as well as their likelihood of persisting to degree completion [3,4,5]. Previous studies show that reducing perceived stress, which financial stress can contribute to, improved sleep quality [63,64]. Further, improving sleep quality led to improved eating behaviors [65,66]. Based on experimental studies noting the causal relationships between perceived stress and sleep quality as well as with sleep quality and dietary behaviors [63,64,65,66], the mediation effect of sleep quality on the relationship between financial stress and dietary risk score is plausible. Additionally, one other study explored the mediation effect of sleep quality on the relationship between food insecurity and obesity and revealed that poor sleep quality, specifically trouble falling asleep and maintaining sleep, mediated the relationship between food insecurity and obesity [67]. Because obesity is associated with undesirable dietary behaviors [16,17], and based on the totality of the evidence, poor sleep quality appears to serve as an intermediate factor that explains the connection between financial stress and dietary risk score.

### 4.3. The Mediation Effect of Short Sleep Duration on the Relationship between Financial Stress and Dietary Risk Score

As hypothesized, the present study found that short sleep duration mediated the relationship between financial stress and dietary risk score among students reported short sleep duration. This finding is consistent with the finding from another study, in which it was noted that very short (≤4 h per night) and short sleep (5 to 6 h per night) duration mediated the relationship between food insecurity and obesity [67]. There are several mechanisms that could plausibly contribute to these relationships. First, stress, including financial stress, disrupts sleep and leads to short sleep duration through stress activates the hypothalamic-pituitary-adrenal (HPA) axis [68]. Stress hormone activation of the HPA axis leads to wakefulness and shortened sleep duration [69]. Second, sleep deprivation or curtailment has been associated with increased neural and behavioral reactivity to positive experiences [70]. For example, eating sweets is an enjoyable experience, and under the situation of sleep deprivation or curtailment, eating sweets becomes more enjoyable than usual, which could lead to overconsumption [71]. Based on the previous causal relationship studies, the previous mediation study, and the findings of the present study, short sleep duration could serve as an intermediate factor that explains why financial stress and overall dietary risk score are connected.

### 4.4. Overall Public Health Messages

Given the results of the present study, strategies for improving sleep quality and duration among higher education students may serve as a potential intervention to reduce risky dietary behaviors when financial stress is high. However, assessing sleep and providing sleep interventions have been overlooked among universities [72,73]. Despite the fact that higher education students have multiple demands on their time, various techniques have been successful in improving sleep outcomes in this population. These strategies include cognitive behavioral therapy for insomnia (CBTi), sleep hygiene education, mindfulness, relaxation, and hypnotherapy [74,75,76,77,78]. Further, one systematic review noted that CBTi was the most effective strategy for sleep improvement compared to other strategies [76], and its online delivery has also been shown to be effective [79]. Under situations like the current pandemic, online CBTi could be a feasible way to help students improve sleep. As both poor sleep quality and short sleep duration mediated the relationship between financial stress and dietary risk behaviors among poor sleepers, improving sleep when financial stress is high may prevent undesirable health behaviors or outcomes, like poor dietary choices or weight gain, among higher education students. Taken together, universities should consider feasible ways to improve the sleep of students in order to improve the health of students in higher education, especially when financial stress is a concern.

The present study suggests that financial stress reduction strategies could also serve as a potential intervention to reduce risky dietary behaviors among U.S. higher education students. Factors contributing to financial stress among higher education students include, not having enough money to participate in the same activities as peers, requiring additional finances to meet unexpected costs, sourcing additional finances from external loans, working more than 12 h per week, living costs, and expecting to have higher amounts of student loan debt at graduation [80,81]. Financial education and counseling are effective strategies to reduce financial stress and improve financial satisfaction among higher education students [82,83,84,85,86], and these strategies aim to help students identify sources and factors that cause financial stress as well as improve financial behaviors [86,87]. One meta-analysis noted that financial literacy is most effective when provided at the time of financial struggle [88]. Given the considerable evidence showing that the majority of the higher education students currently experience financial stress [9], addressing financial literacy at the university level could be an effective way to reduce financial stress and promote overall well-being of higher education students. Thus, university administrators should consider providing financial education and counseling to higher education students.

### 4.5. Strengths and Limitations

There are several strengths to this study. First, the large sample size in the present study provided adequate power to conduct the regression and mediation analyses. Second, using the mediation analysis allowed for the exploration of more complex relationships between financial stress, sleep duration and quality, and dietary risk score. Third, the study included students from three different large universities in the U.S., which strengthens our ability to generalize to students at other universities. Fourth, the study used validated tools for the higher education student population to measure financial stress, sleep, dietary intake, and dietary risk score [35,45,89,90].

There are limitations to the study. First, the study is a cross-sectional study; therefore, it does not allow for the attribution of causality between the variables examined. Further investigations are needed to confirm possible causal relationships. Second, the study was not able to compare the combined mediation effects of poor sleep quality and short sleep duration on the relationships between financial stress and the dietary risk score because sleep quality and duration were measured using different scales and were assessed in two separate models. Standardized path analyses might be considered in the future to overcome this limitation. Third, the universities studied are large, public universities, and in-state tuition is relatively low compared to private institutions; although, there was no stipulation that students completing the surveys had to be in-state students. Fourth, the study did not collect socioeconomic status of the participants, which could be a possible covariate. Fifth, the study was conducted at the beginning of the COVID-19 pandemic, and studies demonstrate that the pandemic has influenced variables that we measured, including increased financial and perceived stress and worsened sleep [91,92]. In the present study, the majority of students indicated increased financial stress as a part of their pandemic experience. Therefore, these relationships should be examined again once financial stress, perceived stress, sleep, and life, in general, more closely resembles the pre-pandemic situation.

## 5. Conclusions

Sleep duration and quality were associated with poorer dietary quality based on intake data from multiple nutrients and food groups. For students who reported insufficient sleep or poor sleep quality, these sleep problems completely mediated the relationship between financial stress and dietary risk scores. To reduce the influence of financial stress on undesirable dietary behaviors, student programming should emphasize both sleep and financial literacy training.

## Figures and Tables

**Table 1:** Demographic, anthropometric, and sleep characteristics.

	U.S. Higher Education Students N = 1280	
	N (%)	(Mean ± SD)
Gender		
Male	308 (24)	-
Female	935 (73)	-
Other	37 (3)	-
Graduate status		
Undergraduate students	951 (74)	-
Graduate students	329 (26)	-
		
Residency status		
Domestic students	1113 (87)	-
International students	167 (13)	-
Age (y)	1266	22.5 ± 4.8
BMI (kg/m^2^)	1273	25.9 ± 6.2
Sleep quality		
Poor sleeper	996 (78)	8.6 ± 3.0
Good sleeper	284 (22)	3.0 ± 1.0
Sleep duration		
Did not meet recommendations	310 (24)	6.0 ± 0.7
Met recommendations	970 (76)	8.2 ± 0.9

Demographic, anthropometric, and sleep characteristics of the study sample. SD = Standard deviation. Poor sleepers were classified as reporting a PSQI score ≥5. “Did not meet sleep recommendations” refers to students who reported nightly sleep duration <7 h. Missing Data: age (N = 1); BMI = body mass index (N = 7); gender (N = 0); graduate status (N = 0); residency status (N = 0).

**Table 2:** Financial stress and its associations with demographic and health characteristics.

Continuous Measures	Mean ± SD	Correlation Coefficient with Financial Stress
Financial stress (USFSA score)	17.4 ± 5.9	---
Age (years)	22.5 ± 4.8	0.067 *
BMI (kg/m^2^)	25.9 ± 6.2	0.216 ***
Perceived stress (PSS-10 score)	21.7 ± 7.1	0.341 ***
Sleep quality (PSQI score)	7.4 ± 3.6	0.325 ***
Sleep duration (h)	7.6 ± 1.3	−0.199 ***
Physical activity level (METs minutes per week)	3330.5 ± 4056.3	0.034
**Categorical Measures**	**Financial Stress** **Mean ± SD**	***p* Value**
Gender		
Male	16.5 ± 5.9 ^a^	0.006
Female	17.6 ± 5.8 ^b^	
Other	18.9 ± 5.5 ^a,b^	
Graduate status		
Undergraduate students	17.5 ± 5.9	0.079
Graduate students	16.9 ± 5.7	
Residency status		
Domestic students	17.6 ± 5.9	0.001
International students	16.0 ± 5.2	

Financial stress as measured by the USFSA according to gender, degree, and residency status. Note that sleep quality was measured using the PSQI, and higher PSQI scores indicate lower sleep quality. * *p* < 0.05; *** *p* < 0.001. Benjamini–Hochberg adjusted *p* values were used for determinations of significance. Means with different superscripts are significantly different, *p* < 0.05. SD = Standard deviation. Missing Data: age (N = 1); BMI (N = 7); gender (N = 0); graduate status (N = 0); residency status (N = 0); perceived stress (N = 0); sleep quality (N = 8); sleep duration (N = 9); physical activity level (N = 115).

**Table 3:** Dietary intake, dietary risk score, and their associations with demographic and health characteristics of U.S. higher education students (continuous variables).

	Outcome Measures										
Continuous Measures	Dairy (cups/d)	Whole Grains (oz/d)	Vegetables (cups/d)	Fruit (cups/d)	Red Meat (times/d)	Processed Meat (times/d)	Fiber (g/d)	Calcium (mg/d)	Total Added Sugar (tsp/d)	Added Sugar from SSB (tsp/d)	Dietary Risk Score
Mean ± SD	1.6 ± 0.6	0.7 ± 0.3	1.3 ± 0.3	0.9 ± 0.4	0.3 ± 0.3	0.2 ± 0.2	15.6 ± 3.2	968.8 ± 207.9	16.3 ± 6.0	6.6 ± 4.1	8.2 ± 2.7
Age (y)	0.018	0.078 **	0.233 **	0.014	−0.021	−0.029	0.148 ***	0.022	0.074 *	0.071 *	−0.062 *
BMI (kg/m^2^)	0.035	−0.070 *	−0.059	−0.100 ***	0.067 *	0.077 **	−0.125 ***	−0.003	0.066 **	0.077 **	0.134 ***
Perceived stress (PSS-10 score)	−0.064 *	−0.079 **	−0.210 ***	−0.115 ***	−0.105 ***	0.004	−0.224 ***	−0.151 ***	0.043	0.020	0.183 ***
Sleep quality (PSQI score)	−0.015	−0.085 **	−0.112 ***	−0.111 ***	−0.050	0.009	−0.192 ***	−0.092 **	0.083 **	0.103 ***	0.164 ***
Sleep duration (h)	−0.018	0.008	−0.024	0.018	−0.041	−0.052	0.024	−0.005	−0.055	−0.099 **	0.015
Physical activity level (METs minutes per week)	0.033	0.067	0.121 ***	0.097 *	0.036	0.056	0.110 ***	0.085 **	−0.042	−0.011	−0.133 ***

Pearson correlation coefficients for the relationships between dietary intake, measured by DSQ, and measured variables. Note: Numbers listed in tables represent correlation coefficient. * *p* < 0.05; ** *p* < 0.01; *** *p* < 0.001; *p* < 0.05 was used to determine significance and Benjamini–Hochberg adjusted p values were used for excluding possible false positive results. All but the associations between physical activity level and whole grains remained significant after the Benjamini–Hochberg false discovery adjustment. SD = Standard deviation. Missing data: age (N = 1); BMI (N = 7); gender (N = 0); graduate status (N = 0); residency status (N = 0); perceived stress (N = 0); sleep quality (N = 8); sleep duration (N = 9); physical activity level (N = 115); dairy (N = 41); whole grains (N = 37); vegetables (N = 37); fruit (N = 37); red meat (N = 4); processed meat (N = 1276); fiber (N = 37); calcium (N = 37); total added sugar (N = 40); total added sugar from SSB (N = 52); dietary behaviors (N = 0).

**Table 4:** Dietary intake, dietary risk score, and their associations with demographic and health characteristics of U.S. higher education students (categorical variables).

Categorical Measures	Dairy (cups/d)	Whole Grains (oz/d)	Vegetables (cups/d)	Fruit (cups/d)	Red Meat (times/d)	Processed Meat (times/d)	Fiber (g/d)	Calcium (mg/d)	Total Added Sugar (tsp/d)	Added Sugar from SSB (tsp/d)	Dietary Risk Score
	M ± SD	M ± SD	M ± SD	M ± SD	M ± SD	M ± SD	M ± SD	M ± SD	M ± SD	M ± SD	M ± SD
Gender											
Male	2.0 ± 0.7	0.7 ± 0.3	1.5 ± 0.4	1.0 ± 0.5	0.4 ± 0.4	0.3 ± 0.3	17.8 ± 3.5	1176.8 ± 249.6	19.3 ± 7.8	8.4 ± 5.1	8.0 ± 2.9
Female	1.5 ± 0.5	0.7 ± 0.3	1.3 ± 0.3	0. 9 ± 0.4	0.3 ± 0.3	0.2 ± 0.2	14.8 ± 2.7	900.3 ± 134.3	15.3 ± 5.0	6.0 ± 3.5	8.2 ± 2.6
Other	---	---	---	---	0.1 ± 0.2	0.2 ± 0.2	---	---	---	---	8.7 ± 2.3
*p* value	<0.001	0.001	<0.001	0.043	<0.001	<0.001	<0.001	<0.001	<0.001	<0.001	0.261
Graduate status											
Undergraduate student	1.6 ± 0.6	0.7 ± 0.3	1.3 ± 0.3	0.9 ± 0.4	0.3 ± 0.3	0.2 ± 0.2	15.3 ± 3.1	964.7 ± 211.8	16.4 ± 6.2	6.7 ± 4.2	8.3 ± 2.7
Graduate student	1.6 ± 0.6	0.7 ± 0.3	1.4 ± 0.4	1.0 ± 0.4	0.3 ± 0.3	0.2 ± 0.2	16.4 ± 3.3	980.7 ± 195.9	16.0 ± 5.4	6.3 ± 3.5	7.7 ± 2.7
*p* value	0.309	0.004	<0.001	0.140	0.460	0.144	<0.001	0.236	0.297	0.080	0.001
Residency status											
Domestic student	1.6 ± 0.6	0.7 ± 0.3	1.3 ± 0.3	0.9 ± 0.4	0.3 ± 0.3	0.21 ± 0.2	15.5 ± 3.1	968.4 ± 206.8	16.4 ± 6.1	6.6 ± 4.0	8.3 ± 2.6
International student	1.6 ± 0.6	0.7 ± 0.3	1.4 ± 0.4	1.0 ± 0.4	0.4 ± 0.4	0.15 ± 0.2	16.1 ± 3.1	917.4 ± 215.8	16.0 ± 6.3	6.8 ± 4.7	7.3 ± 3.1
*p* value	0.734	0.950	0.001	0.201	0.005	0.001	0.019	0.866	0.448	0.527	<0.001

Pearson correlation coefficients for relationships observed between dietary intakes, measured by the DSQ, and demographic measures. M = mean, SD = standard deviation. Missing data: gender (N = 0); graduate status (N = 0); residency status (N = 0); dairy (N = 41); whole grains (N = 37); vegetables (N = 37); fruits (N = 37); red meat (N = 4); processed meat (N = 1276); fiber (N = 37); calcium (N = 37); total added sugar (N = 40); total added sugar from SSB (N = 52); dietary behaviors (N = 0). Note that intake data could not be calculated for a gender other than male and female using the DSQ; therefore, intake data were missing for students who self-identified as “other”.

**Table 5:** Financial stress and its association with dietary intake and dietary risk score.

	Model A			Model B			Model C		
Financial Stress	B (95% CI)	SE B	*p* Value	aB (95% CI)	SE aB	*p* Value	aB (95% CI)	SE aB	*p* Value
Dairy intake (cups/d)	−0.001 (−0.006, 0.005)	0.003	0.842	0.002 (−0.003, 0.007)	0.002	0.392	0.001 (−0.004, 0.007)	0.003	0.616
Whole grains intake (cups/d)	−0.003 (−0.006,−0.0002)	0.002	0.072	−0.003 (−0.006, −0.0001)	0.002	0.047	−0.002 (−0.006, 0.001)	0.002	0.146
Vegetable intake (cups/d)	−0.008 (−0.011, −0.005)	0.002	<0.001	−0.007 (−0.010, −0.004)	0.002	<0.001	−0.006 (−0.009, −0.003)	0.002	<0.001
Fruit intake (cups/d)	−0.007 (−0.011, −0.003)	0.002	<0.001	−0.007 (−0.011, −0.003)	0.002	0.001	−0.005 (−0.009, −0.001)	0.002	0.026
Red meat intake (times/d)	−0.001 (−0.004, 0.002)	0.001	0.442	0.0004 (−0.002, 0.003)	0.001	0.766	0.002 (−0.001, 0.005)	0.002	0.317
Processed meat intake (times/d)	−0.00001 (−0.002, 0.002)	0.001	0.992	0.0003 (−0.002, 0.002)	0.001	0.816	−0.0005 (−0.003, 0.002)	0.001	0.667
Fiber intake (g/d)	−0.091 (−0.121, −0.062)	0.015	<0.001	−0.082 (−0.109, −0.055)	0.014	<0.001	−0.071 (−0.099, −0.042)	0.015	<0.001
Calcium intake (mg/d)	−2.153 (−4.032, −0.274)	0.958	0.025	−0.771 (−2.334, 0.793)	0.797	0.334	−0.896 (−2.551, 0.759)	0.844	0.288
Total added sugar intake (tsp/d)	0.023 (−0.030, 0.076)	0.027	0.395	0.038 (−0.013, 0.089)	0.026	0.140	0.009 (−0.045, 0.063)	0.027	0.746
Added sugar from SSB intake (tsp/d)	0.059 (0.021, 0.097)	0.019	0.002	0.068 (0.030, 0.106)	0.019	<0.001	0.054 (0.014, 0.094)	0.020	0.008
Overall dietary risk score	0.045 (0.019, 0.071)	0.013	0.001	0.042 (0.016, 0.068)	0.013	0.002	0.019 (−0.008, 0.046)	0.014	0.177

Financial stress, as measured by USFSA scores, and the relationship between dietary intake of food groups and nutrients, measured by DSQ, and dietary risk score as measured by STC. B = beta coefficient; 95%CI = 95% confidence intervals; SE = standard error; Ab = adjusted beta coefficient. Model A: unadjusted. Model B: Model A + age, gender, residency status. Model C: Model B + perceived stress.

**Table 6:** Model 1 Mediation Analysis among Students with Poor Sleep Quality.

Variables	B	SE	t	*p* Value
Financial stress → poor sleep quality	0.16	0.02	10.05	<0.001
Poor sleep quality → dietary risk score	0.07	0.03	2.48	0.013
Financial stress → dietary risk score	0.02	0.02	1.12	0.262
Bootstrap	Effect	SE	LL 95%CI	UL 95%CI
Poor sleep quality	0.012	0.005	0.002	0.022

Note: N = 847 in the model.

**Table 7:** Model 3 Mediation Analysis among Students with Good Sleep Quality.

Variables	B	SE	t	*p* Value
Financial stress → good sleep quality	−0.01	0.01	−0.52	0.603
Good sleep quality → dietary risk score	0.23	0.11	2.16	0.031
Financial stress → dietary risk score	0.04	0.02	1.87	0.062
Bootstrap	Effect	SE	LL 95%CI	UL 95%CI
Poor sleep quality	−0.001	0.003	−0.009	0.004

Note: N = 411 in the model.

**Table 8:** Model 2 Mediation Analysis of Students Not Meeting the Minimum Sleep Duration.

Variables	B	SE	t	*p* Value
Financial stress → short sleep duration	−0.02	0.01	−2.04	0.043
Short sleep duration → dietary risk score	−0.69	0.24	2.85	0.005
Financial stress → dietary risk score	0.03	0.03	0.78	0.436
Bootstrap	Effect	SE	LL 95%CI	UL 95%CI
Short sleep duration	−0.01	0.007	−0.026	−0.0003

Note: N = 303 in the model.

**Table 9:** Model 4 Mediation Analysis of Students Meeting the Minimum Sleep Duration.

Variables	B	SE	t	*p* Value
Financial stress → adequate sleep duration	−0.01	0.01	−1.10	0.270
Adequate sleep duration → dietary risk score	0.19	0.09	2.05	0.041
Financial stress → dietary risk score	0.02	0.02	1.45	0.147
Bootstrap	Effect	SE	LL 95%CI	UL 95%CI
Short sleep duration	−0.001	0.001	−0.0043	0.0011

Note: N = 954 in the model.

## Data Availability

The data presented in this study are available on request from the corresponding author. The data are not publicly available due to ongoing analyses.
